# Gut microbiota–mediated tryptophan metabolic dysregulation in the comorbidity of inflammatory bowel disease and autism spectrum disorder: mechanisms and research progress

**DOI:** 10.3389/fimmu.2026.1950055

**Published:** 2026-10-09

**Authors:** Mingyu Lu, Yuxuan Chen, Hengli Zhou, Yingzhuo Lin

**Affiliations:** 1 First Clinical Medical College, Guangdong Medical University, Zhanjiang, China; 2 Department of Traditional Chinese Medicine, Yangjiang People’s Hospital, Yangjiang, China; 3 Department of Gastroenterology, Yangjiang People’s Hospital, Yangjiang, China

**Keywords:** autism spectrum disorder (ASD), gut microbiota, gut-brain axis (GBA), inflammatory bowel disease (IBD), kynurenine, mucosal immunity, serotonin (or 5-hydroxytryptamine 5-HT), tryptophan metabolism

## Abstract

The “microbiota-gut-brain axis” represents a critical link connecting various aspects of intestinal physiology, brain function, and behavioral performance. Among these, tryptophan metabolism abnormalities mediated by gut microbes through the gut-brain axis (GBA) have garnered widespread attention in the study of IBD-ASD comorbidity. This article reviews the current state of research on tryptophan metabolism abnormalities in IBD-ASD comorbidity, with a particular focus on the connection between peripheral and central tryptophan metabolic derangements mediated by gut microbes via the GBA. Furthermore, we summarize and analyze potential therapeutic targets and intervention strategies in this comorbid condition, integrating recent mechanistic studies and clinical evidence to provide a conceptual framework for understanding the potential mechanisms underlying IBD-ASD comorbidity. However, direct evidence from patients with confirmed IBD-ASD comorbidity remains limited at present, and further clinical studies are still needed to test this hypothesis.

## 1 Introduction

Inflammatory bowel disease (IBD) has become a global chronic disorder, characterized by typical pathological alterations and gastrointestinal manifestations such as diarrhea and abdominal pain. The incidence of pediatric IBD is continuously rising worldwide. The highest incidence and prevalence rates are recorded in Northern Europe and North America, whereas Southern Europe, Asia, and the Middle East exhibit relatively low prevalence (1). China harbors one of the largest IBD patient populations globally, with its incidence increasing rapidly year by year. Accordingly, the substantial disease burden of IBD has emerged as a critical public health concern in China (2). Accumulating clinical and experimental evidence has demonstrated a correlation between IBD and autism spectrum disorder (ASD), and an increasing number of clinical cases have documented IBD-ASD comorbidity (3–6).

ASD is a highly heterogeneous neurodevelopmental disorder with onset in early infancy. It is primarily characterized by repetitive stereotyped behaviors, restricted interests, and impaired social communication (7, 8), and is frequently accompanied by multisystem comorbidities (9). In recent years, the global incidence of ASD has continued to rise, with approximately one in every 100 children worldwide diagnosed with ASD (7). The prevalence of ASD among Chinese children is about 1.0%, which is close to the global level (10). ASD has become a priority issue in China’s public health and disability services, requiring urgent attention and effective intervention (11).

Based on the distinct epidemiological characteristics of IBD and ASD, the comorbidity of the two diseases has attracted growing public health attention in China. Meanwhile, such epidemiological features may provide promising research targets for exploring the underlying mechanisms of this comorbidity.

Accumulating evidence indicates that the pathogenesis and clinical manifestations of both IBD and ASD are tightly linked to tryptophan metabolic disturbances (12, 13). Tryptophan dysregulation has been proposed as a pivotal hub, whereby gut microbiota mediate this comorbidity through the gut-brain axis (GBA). On the one hand, it modulates intestinal inflammation and affects intestinal immune and barrier functions; on the other hand, it alters central neurotransmitter activity and triggers neuroinflammation via the blood-brain barrier or vagus nerve.

Current studies regarding IBD-ASD comorbidity predominantly focus on clinical phenotypic summaries and separate mechanistic explorations of individual diseases, while a hierarchical and integrative synthesis labor concerning the pivotal role of tryptophan metabolic dysregulation in comorbid pathogenesis remains insufficient (14–16). The evidence presented in this review is derived primarily from epidemiological associations and preclinical data from animal and *in vitro* models, on the basis of which we propose a hypothetical mechanistic framework for the IBD-ASD comorbidity. This review centers on the mechanisms and research progress of gut microbiota-mediated tryptophan metabolic abnormalities in IBD-ASD comorbidity, with a particular emphasis on key findings and unresolved issues. It proposes an integrative mechanistic framework for underexplored area by proposing a pathway by which gut microbiota mediate comorbidity through the GBA, integrates recent mechanistic and clinical evidence, and offers new perspectives for understanding the specific mechanisms of comorbidity as well as existing research controversies. Ultimately, this review aims to provide theoretical support for future comorbid therapeutic strategies, including tryptophan derivative supplementation and targeted gut microbiota modulation.

The literature search was conducted using the PubMed database with the following English keywords: “inflammatory bowel disease,” “autism spectrum disorder,” “gut microbiota,” “gut-brain axis,” “tryptophan metabolism,” “kynurenine,” “serotonin,” and “5-hydroxytryptamine.” The corresponding Chinese keywords were used to search the China National Knowledge Infrastructure (CNKI), Wanfang Data Knowledge Service Platform, VIP Chinese Science and Technology Periodical Database, and the Chinese Biomedical Literature Service System (SinoMed). The search covered the period from the inception of each database to December 2025. The inclusion criteria were: (1) peer-reviewed original articles and high-quality reviews published in English or Chinese; and (2) studies providing critical evidence supporting the mechanistic framework of this review. The exclusion criteria were: (1) studies with insufficient data or duplicate publications; (2) articles that could not be obtained in full text; and (3) studies of low methodological quality.

## 2 IBD-ASD comorbidity

Epidemiological evidence has demonstrated that patients with IBD may exhibit a higher incidence of neurodevelopmental disorders, such as ASD compared with non-IBD individuals (17). Parental IBD (including its subtypes), particularly in mothers, is potentially causally associated with an increased risk of ASD in offspring (6). Conversely, individuals with ASD are more susceptible to IBD than those without ASD (3, 5). Children diagnosed with ASD also have a significantly higher subsequent risk of developing IBD than healthy controls and the reported baseline incidence of pediatric IBD in the general population (3).

In patients with IBD, studies have confirmed that comorbidity with ASD exacerbates the symptoms and severity of intestinal inflammation (18). In terms of clinical prognosis, patients with such comorbidities achieve poorer outcomes than those diagnosed solely with IBD or ASD. Compared with uncomplicated pediatric UC patients, children with UC combined with ASD exhibit significantly lower Pediatric Ulcerative Colitis Activity Index (PUCAI) scores, accompanied by higher utilization rates of immunomodulators and biological agents. This phenomenon is attributed to the blunted pain perception and insufficient subjective symptom reporting in these children, which result in underestimated clinical scores. Nevertheless, they suffer from more severe actual intestinal pathological lesions, thereby necessitating more intensive pharmacological interventions (18).

These findings indicate that IBD-ASD comorbidity may not be simply a superposition of symptoms of the two disorders, but tends to arise from a bidirectional vicious cycle driven by shared pathogenic mechanisms.

### 2.1 Tryptophan metabolism in IBD and ASD

Tryptophan metabolism plays an essential role in regulating multiple physiological functions of the human body, which primarily involves three pathways: the kynurenine (KYN) pathway, the serotonin pathway, and the microbial metabolic pathway (**Table 1**). In mammals, the vast majority of tryptophan is catabolized through the kynurenine pathway, while a smaller fraction is directed toward serotonin synthesis or directly metabolized by the gut microbiota (19, 20). Although the microbiota-mediated route accounts for a relatively minor share of global tryptophan turnover, it produces a diverse array of bioactive indole derivatives that can exert disproportionately potent effects on host immunity, barrier function, and neuroendocrine signaling via the gut–brain axis. The specific enzymatic steps, tissue distribution, and physiological functions of each of these three pathways—kynurenine, serotonin, and microbial—are discussed in detail in the following sections.

**Table 1:** Tryptophan metabolic pathways, metabolites and physiological functions.

Metabolic pathway category	Branched metabolic pathway	Key enzyme	Metabolites	Physiological function
Tryptophan	Kynurenine pathway	IDO, TDO	N-formylkynurenine, KYN, KYNA, QA, nicotinic acid (precursor of NAD^+^), etc.	Participate in the regulation of immune tolerance Maintain neural homeostasis Generate NAD^+^ involved in redox reactions and cellular repair
	Serotonin pathway	TPH, AADC	5-HTP, 5-HT, 5-HIAA, Melatonin	The central nervous system regulates emotions, appetite and sleep Peripherally regulate gastrointestinal peristalsis and vasoconstriction. Melatonin maintains the circadian rhythm
	Microbial tryptophan metabolism	Bacterial tryptophanase	Indole, IPA, IAA, ILA, IAld	Activate AhR to maintain the intestinal barrier Inhibit inflammation Regulate immunity Participate in the signal transmission of the gut-brain axis

IDO, indoleamine 2,3-dioxygenase; TDO, tryptophan 2,3-dioxygenase; TPH, tryptophan hydroxylase; AADC, aromatic amino acid decarboxylase; KYN, kynurenine; KYNA, kynurenic acid; QA, quinolinic acid; 5-HTP, 5-hydroxytryptophan; 5-HT, 5-hydroxytryptamine; 5-HIAA, 5-hydroxyindoleacetic acid; IPA, indole-3-propionic acid; IAA, indole-3-acetic acid; ILA, indole-3-lactic acid; IAld, indole-3-aldehyde; NAD+, nicotinamide adenine dinucleotide; AhR, aryl hydrocarbon receptor.

#### 2.1.1 Kynurenine pathway and its downstream metabolites

Metabolites derived from the kynurenine pathway, a branch of tryptophan metabolism, play an important role in sustaining neural activity and regulating inflammatory responses. Both the central nervous system and peripheral tissues possess kynurenine metabolism, the precursor of which originates from dietary tryptophan.

In the periphery, the rate-limiting enzymes mediating tryptophan conversion to KYN include TDO and IDO, with IDO being widely expressed in both peripheral and central tissues. TDO is predominantly expressed in the liver, which is the primary organ for KYN production under physiological conditions. Under inflammatory conditions, the pro-inflammatory cytokine IFN-γ upregulates IDO expression in peripheral immune cells, and the resulting increase in plasma KYN mainly originates from these IFN-γ-activated peripheral immune cells (21). The end products of peripheral kynurenine metabolism are quinolinic acid (QA) and kynurenic acid (KYNA). KYNA modulates oxidative and nitrosative stress pathways and reduces the production of inflammatory factors (22). In contrast, the induction and activation of inflammatory pathways by QA are closely associated with the development of intestinal inflammation, and studies have found that QA levels are significantly elevated in patients with IBD (23).

The role of the kynurenine pathway in the central nervous system is primarily reflected through changes in the levels of its metabolites. When an inflammatory response occurs, IDO (indoleamine 2,3-dioxygenase), the rate-limiting enzyme of the kynurenine pathway in the CNS, is activated, leading to the consumption of large amounts of tryptophan to produce KYN. The downstream products generated from KYN each exert distinct physiological effects (24). One of these metabolites, QA, acts primarily by overactivating NMDA receptors in the CNS, leading to neuronal calcium overload and ultimately resulting in excitotoxic injury and neuronal apoptosis (25). In addition, QA can activate microglia and astrocytes, promoting the release of pro-inflammatory cytokines such as TNF-α and IL-6, and generating reactive oxygen species, thereby inducing and exacerbating neuroinflammation and oxidative stress, which further contribute to neural tissue damage (26). Consequently, QA is considered a typical neurotoxic metabolite (26, 27).

In contrast, another downstream metabolite of the kynurenine pathway, kynurenic acid (KYNA), is considered to exert neuroprotective effects in the central nervous system. Excessive activation of excitatory neurotransmitter receptors, including glutamate receptors is known to trigger neurotoxicity, while KYNA can competitively bind to relevant receptors in the central nervous system, restrain the overactivation of excitatory neurotransmitters, relieve neuronal damage and exert neuroprotective effects (28). Besides, KYNA inhibits the secretion of pro-inflammatory cytokines like TNF-α and IL-6, thus mitigating neuroinflammatory responses (19). It also modulates the activity of key enzymes in the kynurenine pathway such as IDO, lowers the synthesis of neurotoxic QA, and maintains metabolic homeostasis to safeguard neural cells (27). Once the synthesis of anti-inflammatory metabolites declines while pro-inflammatory metabolites accumulate, such metabolic imbalance cannot be corrected by physiological regulation, which will trigger central neuroinflammation and further participate in the onset and progression of ASD.

Metabolites of the peripheral kynurenine pathway can cross the blood-brain barrier (BBB) to exert combined effects. However, not all downstream products of the peripheral kynurenine pathway can cross the BBB; only its precursor tryptophan, KYN itself, and 3-hydroxykynurenine (3-HK) can enter the CNS through the BBB, whereas KYNA, QA, TPO, and peripherally expressed IDO are unable to cross the BBB into the CNS. Under inflammatory conditions, pro-inflammatory cytokines can induce peripheral immune cells to upregulate IDO expression in the periphery, leading to elevated plasma KYN levels. KYN can cross the BBB and enter the brain, providing substrate for central synthesis of KYNA and QA, a process that does not require CNS IDO activation. Thus, the peripheral and central kynurenine pathways are primarily interconnected and mutually regulated through fluctuations in KYN levels (21).

#### 2.1.2 Serotonin pathway and its downstream metabolites

As another vital branch of tryptophan metabolism, the serotonin pathway generates metabolites that perform unique physiological regulatory functions in both peripheral tissues and the central nervous system (29). Circulating tryptophan can cross the blood-brain barrier and enter brain tissues. Subsequently, raphe nucleus neurons enrich tryptophan and express metabolic enzymes, among which tryptophan hydroxylase (TPH) acts as the rate-limiting enzyme. Tryptophan is first catalyzed into 5-hydroxytryptophan (5-HTP), which is further transformed into 5-hydroxytryptamine (5-HT, serotonin). Central 5-HT may play an important role in neurodevelopment, even transient disturbance of the serotonin system during developmental stages can induce persistent alterations in brain function and behavior (30). Preclinical data from animal experiments using Tph2-knockout mice have demonstrated that complete depletion of brain 5-HT impairs isolation-induced ultrasonic vocalizations in neonatal pups, a behavioral phenotype relevant to early-life communication deficits observed in ASD. However, such extreme genetic depletion appears to be rare in human ASD patients (31).

In peripheral tissues, tryptophan also acts as a precursor for 5-HT synthesis. Enterochromaffin cells in the intestinal mucosa are the major source of peripheral 5-HT. Within these cells, tryptophan is catalyzed by tryptophan hydroxylase 1 (TPH1), the rate-limiting enzyme, to form 5-hydroxytryptophan (5-HTP), which is further decarboxylated into 5-hydroxytryptamine (5-HT) by aromatic L-amino acid decarboxylase (AADC). Peripherally synthesized 5-HT is secreted by enterochromaffin cells, stored in platelets, and finally metabolized and degraded in the liver (32).

Intestinal microbiota play an important regulatory role in peripheral 5-HT metabolism, and the activity of TPH1 can be modulated by intestinal microenvironmental factors, including dietary components and gut microbial metabolites (33, 34). Experiments conducted in Tph1^-/-^ mice with markedly diminished intestinal 5-HT synthesis have verified that gut-derived 5-HT facilitates the rolling, adhesion and recruitment of neutrophils to acute inflammatory lesions (34, 35).Peripheral 5-HT accounts for over 95% of total serotonin in the human body, and it plays a central role in regulating intestinal peristalsis, secretion and intestinal barrier function within the digestive system (36). Gut microbiota dysbiosis can trigger metabolic disorders of peripheral 5-HT. The gut dysfunction and pathological changes resulting from 5-HT metabolic disturbances may contribute to the initiation and development of IBD.

Central and peripheral 5-HT metabolism do not exist as two independent systems. Due to the presence of the blood-brain barrier (BBB), peripheral 5-HT cannot directly enter the central nervous system (CNS); rather, the peripheral and central 5-HT systems are primarily linked indirectly through tryptophan. Tryptophan in the bloodstream competes with other large neutral amino acids for transporters to enter the CNS, and the ratio of tryptophan to these competing amino acids determines the total amount of tryptophan that enters the brain. Once inside the brain, tryptophan is regulated by the partially saturated enzyme TPH2, which in turn influences central 5-HT synthesis and may further produce behavioral effects (37). Dietary composition can alter the ratio of tryptophan to other neutral amino acids, and gut microbiota can modulate tryptophan metabolism. The GBA may also serve as a key link connecting peripheral and central 5-HT metabolism. The roles of gut microbiota and the GBA will be discussed in detail in the following sections.

### 2.2 Important roles of gut microbiota in tryptophan metabolism

Although the microbial metabolic pathway represents only a minor branch of tryptophan metabolism, both the pathway itself and its downstream products play a significant role in the regulation of physiological functions through tryptophan metabolism. On one hand, tryptophan can be directly metabolized by gut microbiota into indole derivatives such as indole, indole-3-acetic acid (IAA), and 3-indolepropionic acid (IPA), which exert regulatory effects on various physiological activities. On the other hand, gut microbiota can indirectly modulate host tryptophan metabolism through microbial signals that influence the activity of rate-limiting enzymes in the kynurenine and 5-HT pathways, such as IDO and TPH. The specific mechanisms and downstream effects of these two aspects will be detailed in this section.

#### 2.2.1 Microbial tryptophan metabolism and its downstream metabolites

In the microbial metabolic pathway, diverse gut microbiota can ultimately metabolize tryptophan into various indole derivatives, such as indole, indole-3-acetic acid (IAA) and 3-indolepropionic acid (IPA). These indole metabolites exert vital regulatory effects on intestinal motility, intestinal barrier integrity and immune homeostasis.

The composition of gut microbiota involved in this metabolic pathway is diverse. Genomic predictions have revealed that commensal bacteria from multiple phyla in the gut possess the genetic potential to metabolize tryptophan into neuroactive indole metabolites, with indole serving as the precursor for the synthesis of all subsequent indole derivatives (38). Moreover, various Gram-positive and Gram-negative bacteria can metabolize tryptophan via hydroxylases to generate indole-3-carboxaldehyde (IAld), indole-3-acrylic acid (IAAc), and IPA (39). These bacterial groups interact synergistically to form an “intermicrobial metabolic chain,” such that a single end product is not ultimately generated; rather, the types and proportions of metabolites depend on the compositional structure of the gut microbiota. When the composition of the gut microbiota is altered under pathological conditions, the generation of metabolites via the microbial pathway may be affected.

Metabolites produced via this pathway, including IAA, IPA, indole and tryptamine, modulate intestinal physiological activities and further regulate systemic bodily functions (39). Among them, IPA plays an important role in maintaining intestinal barrier integrity (40, 41). It can bind to the aryl hydrocarbon receptor (AhR) expressed on intestinal macrophages, thereby suppressing the secretion of pro-inflammatory cytokines such as TNF-α and IL-6. Disorders of the microbial tryptophan pathway can lower IPA levels and impair intestinal barrier function. Consequently, gut microbiota and endotoxins may translocate into the intestinal lamina propria and may trigger intestinal immune responses. This process may play an important role in the progression from localized intestinal inflammation to systemic inflammation in IBD patients. In addition, ASD patients are highly susceptible to gut dysbiosis, and the resultant intestinal barrier dysfunction may also increase their risk of concurrent digestive disorders. These inferences will be discussed in detail in the following sections.

In contrast to IPA, which plays an important role in intestinal barrier protection and anti-inflammation, IAA exerts a core function in the regulation of immune homeostasis. The aryl hydrocarbon receptor (AhR) is a ligand-dependent transcription factor widely expressed in epithelial cells and immune cells, and its activation effectively modulates immune cell proliferation (42). IAA is a high-affinity ligand for AhR. After binding to AhR on the surface of lymphocytes in the intestinal lamina propria, IAA can induce innate lymphoid cells (ILCs) to differentiate into interleukin-22 (IL-22)-secreting cells and promote IL-22 secretion (43). IL-22 enhances the expression of antimicrobial peptides in intestinal epithelial cells and inhibits the colonization of pathogenic bacteria such as Salmonella and Escherichia coli (44). Additionally, IAA can regulate the Treg/Th17 balance by inducing the differentiation of naive T lymphocytes into regulatory T cells (Tregs) and inhibiting the differentiation of Th17 cells, thereby reducing the production of pro-inflammatory cytokines such as IL-17 (45). The maintenance of immune homeostasis is critical for preventing autoimmune diseases such as IBD and rheumatoid arthritis. Studies using human tissue samples, *in vitro* cell experiments, and animal models of colitis have demonstrated that AhR can directly transactivate intestinal epithelial Alpha-defensin 1, thereby suppressing intestinal inflammation by restoring gut microbiota homeostasis and enhancing mucosal antimicrobial defense. Notably, this pathway is downregulated in Crohn’s disease and colitis, and activation of the AhR-Alpha-defensin 1 axis has been shown to ameliorate dysbiosis and alleviate colitis (46). Meanwhile, immune deficiencies may exist in the majority of ASD patients (47), and IAA can effectively alleviate central nervous system inflammation through AhR activation (48).

#### 2.2.2 Indirect regulation of kynurenine and 5-HT pathways by gut microbiota

In addition to directly regulating physiological functions through the microbial pathway of tryptophan metabolism, gut microbiota may also exert indirect regulatory effects on the kynurenine and serotonin pathways via microbial signals.

Gut microbiota may influence the kynurenine and 5-HT pathways by modulating the activity of their rate-limiting enzymes. With respect to the peripheral 5-HT pathway, studies have shown that Limosilactobacillus mucosae combined with Ligilactobacillus ruminis can synthesize 5-HT when 5-HTP is provided as a substrate (49). In addition, certain spore-forming commensal gut bacteria can influence 5-HT biosynthesis by affecting the transcription and expression of host TPH1 and monoamine oxidase (20, 33). They can also indirectly modulate 5-HT production by influencing the synthesis and metabolism of short-chain fatty acids, tryptamine, and bile acids (37, 50). Moreover, beyond the peripheral indirect regulation, gut microbiota may also influence central 5-HT metabolism via the GBA, thereby modulating 5-HT production in raphe nucleus neurons (37).

The crosstalk between the microbial pathway and other metabolic branches exists not only in peripheral tissues but also in the central nervous system. Microbially derived indole-3-aldehyde (IAld) is capable of suppressing the activity of central TPH2, thereby lowering 5-HT synthesis in raphe nucleus neurons (50).Existing studies have revealed that indole-3-carboxylic acid (ICA), a downstream metabolite of the microbial tryptophan metabolism, can bind to the active site of IDO1 during kynurenine metabolism. It exerts competitive inhibitory effects to downregulate IDO1 activity and reduce the generation of kynurenine pathway metabolites. Such modulatory effects can relieve inflammation and neuronal injury induced by excessive activation of the kynurenine pathway (51).

Gut microbiota not only serve as an integral component of the three major tryptophan metabolic pathways, directly regulating various physiological functions, but also act as a hub that indirectly modulates the kynurenine and 5-HT pathways, collectively maintaining tryptophan metabolic homeostasis under physiological conditions. Numerous studies have confirmed that significant tryptophan metabolic disturbances exist in both patients with IBD and those with ASD. Based on these findings, we speculate that gut microbiota dysbiosis may lead to an imbalance in tryptophan metabolism, which may represent a contributing factor that promotes the initiation and progression of both IBD and ASD.

#### 2.2.3 Important role of gut microbiota in regulating central and peripheral tryptophan metabolic balance via the gut-brain axis

The effects of gut microbiota are not confined to the intestinal tract. Numerous studies have confirmed that gut microbiota dysbiosis is commonly detected in patients with various central nervous system diseases. In recent years, most studies have demonstrated that the gut-brain axis (GBA) plays a key role in mediating the crosstalk between intestinal disorders and central nervous system dysfunction.

GBA refers to a bidirectional regulatory network that connects the intestine and the central nervous system via humoral, immune and neural signals, in which gut microbiota exert core regulatory functions. On the one hand, the intestine can modulate central inflammatory responses and physiological activities in an ascending manner. Gut microbial metabolites can cross the intestinal barrier into the bloodstream, penetrate the blood-brain barrier and regulate central physiological functions. They can also transmit signals to the central nervous system through the enteric nervous system, such as the vagus nerve, thereby affecting the synthesis of central neurotransmitters and relevant biological activities. Studies have proven that intestinal enteroendocrine cells can sense tryptophan-derived microbial metabolites, activate intestinal vagal nerve endings, and convey microbial-derived signals to the central nervous system (52). On the other hand, abundant animal experiments have demonstrated that the central nervous system can in turn regulate the composition of gut microbiota, intestinal barrier permeability and intestinal peristalsis through diverse pathways (53).

Many studies imply that gut microbiota may act as a core hub in the regulation of tryptophan metabolic balance via the GBA. Relevant animal experiments have demonstrated that changes in the composition and abundance of gut microbiota may regulate immune system function, carbohydrate metabolism, and energy metabolism, thereby affecting communication within the GBA in mice (54). Specific metabolites produced by gut microbiota, which serve as vital components of the microbial tryptophan metabolism, may modulate the central kynurenine pathway and serotonin pathway through the GBA (55). For instance, IPA and indole, as lipid-soluble metabolites, have been shown to cross the blood-brain barrier and bind to AhR expressed on central microglia, upregulate the expression of kynurenine aminotransferase (KAT), promote the secretion of neuroprotective KYNA, and thereby inhibit inflammation in the central nervous system (48).

Animal experiments have confirmed that the central nervous system can release central-derived 5-HT via vagal nerve endings in a top-down manner. Such 5-HT acts on the intestine to promote the expression of metabolic enzymes in gut microbiota, thereby increasing the production of tryptophan metabolites and regulating intestinal physiological activities (56).

Based on animal experimental evidence, we speculate that the interaction between the central nervous system and gut microbiota via the gut-brain axis might form a bidirectional cycle that regulates tryptophan metabolic balance, which may play an important role in maintaining normal physiological activities of the intestine and central nervous system. Given the vital function of the gut-brain axis in balancing tryptophan metabolism, it can be inferred that abnormal tryptophan metabolism in either the central nervous system or the intestine can transmit pathological changes to the other end through this axis, triggering pathological disorders on both sides and forming a vicious cycle. This may be one of the potential mechanisms underlying IBD-ASD comorbidity.

### 2.3 Association between gut microbiota–mediated tryptophan metabolic disturbances and the pathogenesis of IBD

IBD is a typical inflammatory disease. Both local intestinal manifestations and systemic symptoms are essentially manifestations of inflammatory activity. There are numerous studies and hypotheses concerning the pathogenesis of IBD. Some scholars hold that genetic susceptibility plays a vital role in its onset, while others elaborate on its pathogenic mechanism from the perspectives of immune imbalance, environmental factors, and gut microbiota dysbiosis (57, 58). In recent years, numerous studies have identified tryptophan metabolic disturbances in IBD patients, and animal experiments have explored the potential involvement of tryptophan metabolism in the pathogenesis of IBD in animal models. The results of these animal studies are suggestive of possible underlying mechanisms that might be relevant to human IBD.

Numerous animal studies have confirmed the vital role of tryptophan and its metabolites in the pathogenesis and prognosis of IBD. Relevant findings have shown that the administration of tryptophan and some of its metabolites can alleviate the severity of colitis in mouse models. Another animal experiment has revealed that dietary tryptophan depletion increases the susceptibility of mice to colitis (12).

Based on the above introduction to the tryptophan metabolic pathways and their downstream products, it is evident that the metabolic balance formed by the three major tryptophan metabolic pathways plays an important role in maintaining normal intestinal physiological functions, intestinal barrier integrity and intestinal immunity. Imbalance among these three pathways may directly or indirectly triggers the occurrence and progression of chronic intestinal inflammation. The intestine is not an isolated organ, and a series of intestinal inflammatory responses caused by abnormal tryptophan metabolism may develop into systemic inflammation through the GBA, which may be one of the important pathological foundation for the onset of IBD.

Some clinical studies suggest that, compared with healthy people, many patients with IBD suffer from gut microbiota dysbiosis, and intestinal microorganisms may play a core role in the pathogenesis of IBD (59). However, it remains unclear in current research whether gut microbiota dysbiosis precedes the onset of IBD or vice versa. Integrating various lines of evidence, it appears that gut dysbiosis plays a significant role in both the pathogenesis and the clinical outcomes of IBD. When gut microbiota dysbiosis occurs, the composition of gut microbiota may change, beneficial bacteria producing tryptophanase and hydroxylases decrease, and microbial derived tryptophan metabolites such as IPA and IAA are also reduced accordingly (60).

Given the above introduction to the downstream metabolites and their effects, it appears that IPA may play a crucial role in maintaining intestinal barrier function. It can promote the expression of tight junction proteins by activating the adenosine monophosphate-activated protein kinase (AMPK) pathway. Reduced IPA level widens the gaps between intestinal epithelial cells and markedly elevates intestinal permeability. Relevant studies have verified that IPA may serve not only as a biomarker for active ulcerative colitis but also as an indicator for disease remission. According to research, serum indole and IPA levels are significantly decreased in mice with active colitis (P < 0.05). and such IPA deficiency tends to return to normal in mice with ulcerative colitis in the remission stage (40).

We hypothesize that when intestinal permeability increases, gut microbiota and endotoxins may be prone to translocate into the lamina propria, which may be associated with the activation of intestinal immune responses and directly induce mucosal erosion and ulceration (42, 44). IAA, another microbially derived tryptophan metabolite, is considered pivotal for maintaining immune homeostasis. Translocated pathogenic bacteria and endotoxins can be sensed by dendritic cells and macrophages, potentially triggering innate immune activation and robust secretion of pro-inflammatory cytokines, including IL-1β, IL-6, TNF, and IL-23, thereby eliciting local inflammation and inflammatory overload. IAA is thought to serve as a high-affinity ligand for the aryl hydrocarbon receptor (AhR). Microbiota dysbiosis reduces the production of this protective metabolite, which could lead to AhR pathway inactivation, disrupted immune homeostasis, and subsequent impairment of the downstream IL-10-mediated anti-inflammatory cascade. Aberrantly activated NK cells, γδ T cells, and innate lymphoid cells (ILCs) may secrete inflammatory cytokines such as IFN-γ, IL-17, and IL-13, further amplifying intestinal inflammatory responses (61).

Beyond excessive innate immune activation, antigens from pathogenic bacteria may stimulate dendritic cells to initiate adaptive immunity. Naive CD4^+^ T cells differentiate into distinct lineages—Th1, Th2, and Th17—under specific cytokine milieus, with particular attention to the imbalance between Th17 and regulatory Treg cells. Disrupted immune homeostasis resulting from both innate and adaptive immune activation appears to be associated with persistent and recurrent intestinal inflammation. As outlined above, IAA, a critical microbially derived tryptophan metabolite, is thought to be required for AhR activation and modulation of the Th17/Treg balance. Based on the above evidence, we propose that gut microbiota dysbiosis promotes intestinal inflammation and immune imbalance, forming a vicious cycle that may represent a potential link in the initiation and progression of IBD and ASD.

Some clinical studies analyzing plasma samples from patients with IBD have revealed that serum tryptophan levels are negatively correlated with disease activity, and that patients with CD and UC have significantly elevated serum levels of the pro-inflammatory metabolite QA and decreased levels of the anti-inflammatory metabolite KYNA (28, 62). These findings suggest that tryptophan deficiency may promote the development or exacerbate disease activity in IBD.

Based on the above introduction to the kynurenine pathway, it is evident that various metabolites in the kynurenine pathway also exert important effects on the pathogenesis of IBD and may contribute to disease aggravation in IBD patients. As mentioned above, pro-inflammatory QA and anti-inflammatory KYNA in the kynurenine pathway maintain a dynamic balance under normal physiological conditions. Gut microbiota dysbiosis induces an intestinal inflammatory state, which might greatly activate IDO1, the rate-limiting enzyme of the kynurenine pathway. Most intestinal tryptophan is diverted into the kynurenine pathway (63), leading to reduced substrates for the branch producing anti-inflammatory and neuroprotective KYNA and decreased corresponding branch metabolites. The body fails to antagonize the pro-inflammatory and neurotoxic effects of KYN and QA, which may further aggravate intestinal inflammatory responses.

In addition to the kynurenine pathway, some clinical studies have conducted comparative analyses of colonic mucosal tissues and peripheral blood samples from patients with Crohn’s disease (CD), a subtype of IBD, and healthy controls. These studies found that CD patients exhibit increased mucosal 5-HT synthesis and reduced reuptake, accompanied by enrichment of the 5-HTTLPR S genotype and elevated circulating 5-HT levels. The enhanced 5-HT signaling may contribute to intestinal inflammation in CD while (64).

As outlined above regarding the 5-HT pathway, enterochromaffin cells in the intestine are the primary sites for synthesis and release of gut-derived 5-HT. Integrating this clinical evidence with the known 5-HT metabolic pathway, we hypothesize that the pathogenesis of IBD may be associated with aberrant activation of the 5-HT pathway. When intestinal mucosal inflammation occurs, it may stimulate the proliferation of enterochromaffin cells, upregulate the expression of TPH1 (the key enzyme for 5-HT synthesis), and downregulate the expression of 5-HTT (the key transporter for 5-HT clearance), accompanied by increased expression of the pro-inflammatory cytokine interleukin-13 (IL-13). These changes ultimately lead to elevated intestinal 5-HT levels. Elevated 5-HT may in turn exacerbate intestinal inflammation and trigger IBD-related symptoms such as intestinal dysmotility and pain (64).

Synthesizing relevant clinical studies and animal experiments, we speculate that the combined effects of dysregulation of microbial tryptophan metabolism, immune imbalance, hyperactivity of the kynurenine pathway, and disturbances in the 5-HT pathway may ultimately contribute to the onset and progression of IBD.

We found that one common feature of the disturbances in the kynurenine and 5-HT pathways is that their metabolic abnormalities may further induce gut microbiota dysbiosis. In turn, gut microbiota dysbiosis also serves as a cause of immune imbalance and abnormalities in these two pathways. This may create a mutually reinforcing vicious cycle among these pathological alterations.

However, there is currently no clear evidence establishing the temporal sequence or causal relationships among these events; perhaps future basic research and clinical studies will address these questions.

Given that the GBA serves as a bridge connecting the gut and the central nervous system (CNS), we speculate that GBA-mediated signaling may also be a key factor linking abnormal tryptophan metabolism in the intestinal environment with that in the CNS. If the CNS mounts stress responses to abnormal clinical symptoms under pathological conditions and transmits stress signals back to the gut via the GBA, thereby intensifying intestinal pathological activities, then abnormal tryptophan metabolism in the gut could lead to aberrant CNS physiological activities through the GBA, which might in turn promote the development of CNS disorders characterized by tryptophan disturbances, such as ASD.

### 2.4 Association between gut microbiota–mediated tryptophan metabolic disturbances and the pathogenesis of ASD

Numerous studies have found that patients with ASD exhibit significant tryptophan metabolic disturbances, manifested as decreased tryptophan levels in plasma and urine (13), increased IDO1 activity (65), and elevated whole-blood 5-HT levels (66), which may be an important factor driving the occurrence and progression of ASD.

Most patients with ASD suffer from gut microbiota dysbiosis, such as reduced microbial diversity and decreased abundance of beneficial bacteria. Studies have found that changes in gut microbiota composition are correlated with specific behavioral characteristics related to ASD (67). Based on various studies, we infer that gut microbiota dysbiosis may act as a permissive factor that, together with disturbances in tryptophan metabolism and immune dysregulation, may predispose the host to the development of ASD.

Reduced gut microbiota diversity and insufficient abundance of beneficial bacteria may indicate a decrease in protective indole metabolites such as IPA and IAA. A clinical study performing global metabolomic profiling on 231 plasma and 97 fecal samples from children with ASD and typically developing controls in a large cohort revealed that the fecal levels of various indole metabolites are significantly negatively correlated with the severity of ASD (68). Based on the above analysis of the microbial metabolic pathway, we infer that the reduction of pivotal protective products IPA and IAA may lead to increased intestinal permeability, and that translocation of gut microbiota and endotoxins into blood circulation may contribute to systemic inflammation in ASD. Systemic inflammation is a low-grade chronic inflammation. The pathological inflammatory characteristics of ASD patients are manifested as elevated levels of pro-inflammatory factors such as TNF-α, IL-6, and IL-17 in peripheral blood (69, 70). These inflammatory factors may activate rate-limiting enzymes in tryptophan metabolic pathways, such as IDO1 in the periphery, thus potentially further amplifying inflammatory responses (65).

We speculate that gut microbial metabolites may drive systemic inflammation in individuals with ASD, and in addition, their deficient production may also be associated with the pathogenesis and shapes clinical manifestations of ASD. A 2025 cross-sectional case-control observational study first clarified how interactions between gut metabolites and brain activity modulate autistic symptoms. This work verified differences in tryptophan-related fecal metabolites between the ASD cohort and neurotypical (NT) controls. Moreover, brain regions and neural activity implicated in interoceptive processing, vagal function, aversive emotion processing, and social-emotional processing exhibited significant correlations with autistic symptoms (71).

As outlined previously, gut microbial metabolites are essential for sustaining immune homeostasis. Notably, IAA mitigates central nervous system inflammation via AhR activation (48). Preclinical studies demonstrate that gut dysbiosis and lowered IPA levels reduce IPA entry into the hippocampus through peripheral circulation, inhibiting the IPA/AhR/NF-κB signaling cascade. This event triggers excessive microglial activation and aberrant hippocampal synaptic pruning, culminating in the emergence of ASD-like behavioral phenotypes (72). Furthermore, animal experiments reveal that the commensal bacterium Faecalibacterium modulates indole–AhR signaling and ameliorates autism-related behavioral deficits in mice (73).

On the other hand, a study has found that microdeletion of chromosome 16p11.2 (16p11.2^+/−^) is a common genetic factor associated with ASD and other neurodevelopmental disorders. IPA supplementation can significantly reverse behavioral and neurological symptoms in animal models with the 16p11.2^+/−^ phenotype (74), which indirectly confirms the vital role of gut microbial metabolites in the pathogenesis of ASD.

From the foregoing, under pathological conditions, the imbalance of the microbial tryptophan metabolism may induce abnormal metabolism of the kynurenine pathway. We speculate that the occurrence of systemic inflammation and neuroinflammation may also lead to the abnormal activation of IDO1 and hepatic tryptophan 2,3-dioxygenase (TDO) (65). Numerous studies have demonstrated significant differences in the levels of various metabolites between children with ASD and normally developing controls. Specifically, patients with ASD show markedly higher plasma KYN levels, decreased KYNA levels, and an elevated KYN/KYNA ratio, as well as increased levels of neurotoxic QA and cytokine IL-6 compared with controls (69–71, 75). Excessive QA production causes overactivation of N-methyl-D-aspartate (NMDA) receptors, which further triggers neuronal calcium overload and results in oxidative stress damage and neuronal apoptosis (76). Such alterations in metabolite contents may weaken neuroprotection and aggravate neurotoxicity during developmental stages, which is highly likely to induce the onset and progression of ASD.

When dysregulation of microbial tryptophan metabolism triggers systemic inflammation and impairs intestinal barrier function, and in the context of systemic inflammation, metabolic imbalance of the kynurenine pathway further might lead to neuroinflammation and various cognitive and developmental disorders, metabolic abnormalities of the 5-HT pathway also form chain reactions with the other two tryptophan metabolic branches. The combined effects of these metabolic disturbances might play an important role in the onset and progression of ASD.

Current studies suggest that patients with ASD may exhibit significantly elevated serum 5-HT concentrations accompanied by central 5-HT deficiency. Clinical studies have shown that children with ASD have higher peripheral serum 5-HT and 5-HT transporter levels than typically developing children. Increased peripheral serum 5-HT and 5-HT transporter levels are significantly positively correlated with core ASD symptoms and disease severity, and negatively correlated with neurodevelopment (77, 78).

In contrast to the markedly elevated peripheral serum 5-HT levels in ASD patients, animal experiments using ASD mouse models have demonstrated that insufficient central 5-HT disrupts the excitation-inhibition balance in the neocortex, as well as sensory processing and social function, and that restoring central 5-HT levels in adult mice can partially reverse ASD-related symptoms (79). Animal experiments have also suggested that the expression of TPH2, the rate-limiting enzyme for central 5-HT synthesis, may be suppressed and its activity reduced (31). Notably, these findings are derived from animal models and can only provide suggestive evidence for the pathogenesis of ASD; definitive conclusions await further validation in human studies. As a key neurotransmitter, central 5-HT regulates neuronal migration, synaptic pruning, and neural circuit formation, and influences the connectivity among the prefrontal cortex, amygdala, and hippocampus—brain regions critically involved in social cognition and emotional regulation. Accordingly, the social and cognitive dysfunction observed in ASD patients appears to be closely associated with central 5-HT deficiency (79, 80).

Current studies on the role of 5-HT in the pathogenesis of ASD mainly focus on its involvement in neurodevelopmental abnormalities, and research also confirms that decreased intestinal 5-HT contributes to gastrointestinal symptoms in ASD patients. A mouse experiment simulating ASD showed that the 5-HT level in the small and large intestinal mucosa decreased by 50%, and its content was correlated with the abundance of certain bacterial taxa (20, 81). As mentioned above, 5-HT is essential for regulating gastrointestinal motility (36). Therefore, we infer that 5-HT metabolic disturbances may be associated with the comorbidity of IBD and ASD.

Based on the above-mentioned association between tryptophan metabolic disturbances and the pathogenesis of ASD, we propose that gut microbiota dysbiosis may act as a permissive factor in the pathogenesis of ASD, disrupting immune homeostasis and promoting both systemic and neuroinflammation in patients with ASD. Together with the pathological changes resulting from metabolic abnormalities in the kynurenine and 5-HT pathways, these combined effects might ultimately contribute to the development of ASD.

### 2.5 The important role of gut microbiota–mediated tryptophan metabolic disturbances in the comorbidity of IBD and ASD

Based on the various arguments summarized above, we propose that gut microbiota–mediated tryptophan metabolic disturbances may play an important role in the IBD-ASD comorbidity. As illustrated in **Figure 1**, gut microbiota regulates tryptophan metabolism to mediate gut-brain axis dysfunction in IBD-ASD comorbidity.

**Figure 1:**
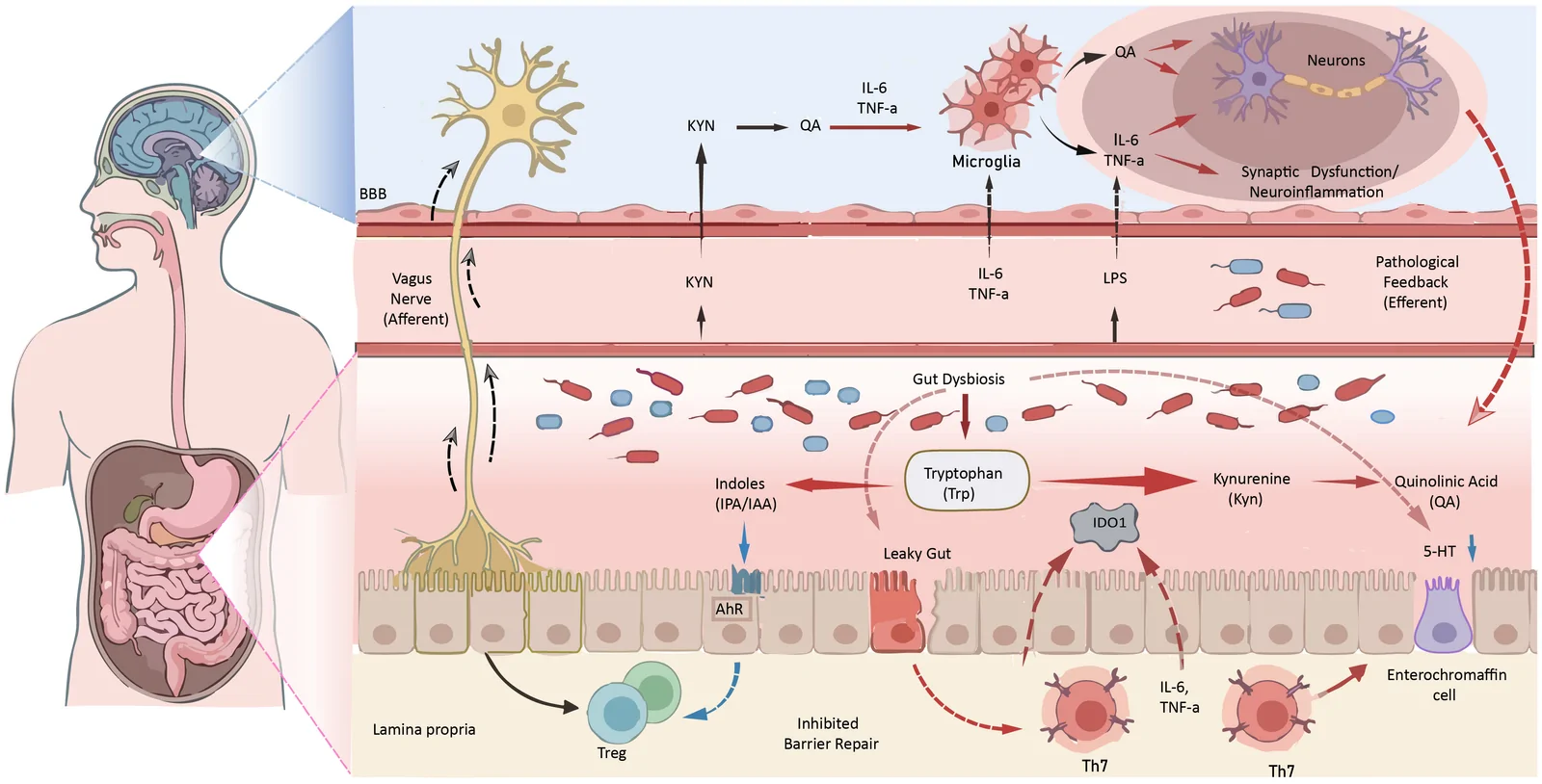
Schematic diagram of the mechanism of gut microbiota-mediated tryptophan metabolic abnormalities in the IBD-ASD comorbidity. The mechanistic connections depicted are derived from evidence demonstrated separately in IBD cohorts, ASD cohorts, and/or experimental models. Direct evidence from patients with confirmed IBD‑ASD comorbidity is currently limited; this model is proposed as an integrative conceptual framework to synthesize current evidence and generate testable hypotheses. Arrow coding denotes levels of supporting evidence: solid lines with solid arrowheads indicate causal mechanistic evidence obtained from in‑vitro and animal experimental models; dashed lines with solid arrowheads represent observational associations identified from human IBD or ASD cohorts without definitive causal proof; dashed lines with hollow arrowheads denote hypothetical inferred links assembled from disjoint independent evidence sets. Solid arrows indicate activation or promotion effects; black arrows represent molecular transport or metabolic transformation processes. Peripheral quinolinic acid (QA) cannot cross the blood‑brain barrier (BBB); only circulating kynurenine (KYN) crosses the BBB to serve as the substrate for central QA synthesis. Trp, Tryptophan; Kyn, Kynurenine; QA, Quinolinic acid; 5‑HT, 5‑hydroxytryptamine; IDO1, Indoleamine 2,3‑dioxygenase 1; AhR, Aryl hydrocarbon receptor; LPS, Lipopolysaccharide; IL‑6, Interleukin‑6; TNF‑α, Tumor necrosis factor‑α; BBB, Blood‑brain barrier.

Under physiological conditions, the diversity and relative abundance of gut microbiota are maintained in a dynamic balance. Through interactions with the kynurenine pathway and the serotonin pathway, the gut microbiota coordinately regulate the homeostasis of the digestive and central nervous systems, thereby maintaining normal physiological functions of the host.

Although the microbial pathway accounts for only a small proportion of tryptophan metabolism, its regulatory effects on physiological functions cannot be ignored. Patients with ASD have a significantly higher risk of gut microbiota dysbiosis than non-ASD individuals. In light of recent studies and hypotheses on ASD and IBD, animal experiments have established models of environmental risk factors for ASD and demonstrated that maternal prenatal exposure to such factors can induce alterations in the abundance of various gut microbial taxa in offspring. Moreover, this microbial dysbiosis may directly affect host health status through metabolic changes and the serotonin pathway (82, 83). Studies have also shown that many IBD patients develop gut microbiota dysbiosis compared with healthy individuals, and gut microbiota may play a core role in the pathogenesis of IBD (59). We reasonably hypothesize that gut microbiota dysbiosis may represent a permissive factor that contributes to the pathogenesis of IBD-ASD comorbidity, although the temporal sequence and causality remain to be established. We reasonably hypothesize that the combined pathological effects resulting from gut microbiota dysbiosis, along with metabolic disturbances in the kynurenine and 5-HT pathways and immune homeostasis imbalance, may have substantially contributed to the onset and progression of IBD-ASD comorbidity.

When gut microbiota dysbiosis develops, the microbial production of protective metabolites such as IPA and IAA via tryptophan metabolism declines, which may lead to intestinal barrier injury and increased intestinal permeability. Subsequent endotoxin translocation may provoke intestinal inflammation and immune responses, and may also lead to insufficient activation of the AhR pathway. The resulting persistent mucosal immune disorders may predispose to the development of IBD (42, 44, 68). Moreover, excessive expansion of pathogenic bacteria may further accelerate the onset and progression of intestinal inflammation. In turn, aggravated intestinal inflammation feeds back on the gut, alters microbial composition and worsens dysbiosis, thereby forming a vicious cycle (84). Intestinal inflammation exacerbated by gut microbiota dysbiosis may also represent an important pathological process contributing to IBD development and the induction of systemic inflammation in ASD.

In response to inflammatory stimuli, the rate-limiting enzyme IDO in the kynurenine pathway is activated, leading to aberrant activation of this pathway and a shift of tryptophan metabolism toward the kynurenine pathway (69, 70). 5-HT production by intestinal enterochromaffin cells is reduced, and this decrease in intestinal 5-HT contributes to the occurrence and aggravation of gastrointestinal symptoms (6, 36, 81). Suppression of the microbial tryptophan pathway results in reduced production of protective indole metabolites, while hyperactivity of the kynurenine pathway increases the production of the pro-inflammatory metabolite QA (71, 76, 78). The combined alterations in these metabolite levels may lead to progressive impairment of intestinal barrier function and increased intestinal permeability, thereby providing conditions for gut microbiota translocation and pathogen invasion, which serve as important prerequisites for the development of IBD and the induction of systemic inflammation in ASD.

Studies have confirmed that children with ASD and comorbid gastrointestinal symptoms show a tendency toward immune imbalance and impaired intestinal barrier function compared with typically developing children (85). Plasma levels of innate cytokines such as IL-1α and TNF-α, as well as adaptive cytokines such as IL-13, are increased in ASD children with gastrointestinal symptoms compared with those without such symptoms. Compared with all other groups, ASD children with gastrointestinal symptoms have elevated plasma levels of the mucosa-associated cytokine interleukin-15 (IL-15). In contrast, levels of the regulatory cytokine interleukin-10 (IL-10) are decreased in ASD children with gastrointestinal symptoms, suggesting a pro-inflammatory/regulatory imbalance in these patients (86). It should be noted that ASD children with gastrointestinal symptoms are not equivalent to those with IBD-ASD comorbidity. However, the presence of gastrointestinal symptoms in ASD children often implies intestinal mucosal barrier damage and a persistent inflammatory state in the gut. As discussed above, intestinal mucosal barrier damage and persistent intestinal inflammation may play an important role in the development of IBD. Based on this, we speculate that ASD children with gastrointestinal symptoms may be more susceptible to comorbid IBD.

Following tryptophan metabolic disturbances that cause intestinal barrier damage, intestinal immune homeostasis may further be disrupted. As mentioned above, indole metabolites generated by microbial tryptophan metabolism are closely associated with intestinal immune function. Studies have confirmed that ASD patients may have immune regulation defects, which can lead to differences in inflammatory T-cell subsets, a phenomenon that may be linked to the comorbidities observed in this population (46). When the microbial tryptophan metabolism is suppressed, the production of IAA, a high-affinity AhR ligand, is reduced, thereby weakening its ability to promote the differentiation of regulatory T cells and suppress the release of pro-inflammatory cytokines. Consequently, the Th17/Treg balance shifts toward a pro-inflammatory direction. Overactivation of the kynurenine pathway can induce immunosuppressive compensatory responses in intestinal immune cells; however, prolonged activation leads to local intestinal immune dysfunction, failure to effectively clear pathogens, and may eventually lead to chronic intestinal inflammation. Abnormal local 5-HT levels can activate 5-HT receptors on intestinal immune cells, promoting the release of pro-inflammatory cytokines such as IL-6 and TNF-α, which may further exacerbate intestinal inflammation (87).

Therefore, when gut microbiota–mediated tryptophan metabolism is disrupted, the alterations—both increases and decreases—in metabolite levels across the three major metabolic pathways give rise to a series of pathological changes. These changes do not exist in isolation, nor are they a mere superposition of individual pathological manifestations. As discussed above, dysbiosis resulting from microbial pathway imbalance, dysregulated anti-inflammatory/pro-inflammatory signaling, and impaired immune homeostasis caused by kynurenine pathway imbalance and gastrointestinal symptoms attributable to 5-HT pathway disturbances all constitute important factors predisposing to IBD. In the central nervous system, the “leaky gut” resulting from gut microbiota dysbiosis may facilitate the entry of endotoxins and other harmful substances into the circulation, which may further trigger systemic inflammation. Systemic inflammation may induce upregulation of IDO activity in the CNS, leading to an imbalance between the neuroprotective metabolite KYNA and the neuroinflammatory metabolite QA. Meanwhile, the expression of TPH2, the rate-limiting enzyme for central 5-HT synthesis, appears to be suppressed, with reduced enzyme activity. The combined effects of these pathological alterations may also represent important factors contributing to the onset and progression of ASD.

Accumulating evidence indicates that pathological changes in the digestive and central nervous systems do not occur in isolation, nor are they independent of each other. The role of gut microbiota is not limited to triggering the separate onset of IBD or ASD; rather, the GBA serves as a critical bridge linking the two systems via the gut microbiota. Furthermore, this review proposes that gut microbiota, through the GBA, may connect the tryptophan metabolic disturbances occurring in both systems, ultimately contributing to the development and progression of IBD-ASD comorbidity.

The GBA orchestrates bidirectional communication between the gut and the central nervous system (CNS), enabling ascending transmission of intestinal signals via neural and humoral pathways, as well as descending modulation of gut functions by the CNS. A 2023 multi-omics study confirmed that ASD metabolic profiles converge along gut-brain pathways and identified associations between specific microbial taxa and ASD-related diet and immunity; fecal microbiota transplantation (FMT) studies further suggest that certain microbes may influence ASD symptoms (88–90). We postulate that the GBA serves as a critical bridge in the comorbidity of ASD and IBD (91).

In summary, we infer that the gut microbiota, acting as a central hub via the GBA, may link central neuroinflammation in ASD with pathological changes induced by gut microbiota dysbiosis. Immune dysregulation serves as both a trigger and an amplifier in IBD-ASD comorbidity, thereby closing the loop of these pathological processes. The continuous mutual reinforcement between local intestinal inflammation and central neuroinflammation may further accelerate the onset and progression of IBD-ASD comorbidity.

## 3 Treatment

Currently, there are well-established clinical treatment protocols for both IBD and ASD. However, specific intervention studies targeting patients with IBD-ASD comorbidity remain extremely limited. This section briefly outlines the established clinical therapeutic strategies for IBD and ASD that have been tested in clinical trials and applied in practice, with a particular focus on experimental intervention strategies relevant to the mechanistic framework proposed in this review. It should be emphasized that the latter are still in preclinical or early-stage clinical exploration and have not yet been directly applied to large-scale treatment of patients with comorbid conditions; their safety and efficacy require further validation.

### 3.1 Established therapies for IBD and ASD

For intestinal inflammation in inflammatory bowel disease (IBD), 5-aminosalicylic acid preparations are the first choice for mild ulcerative colitis (UC). Glucocorticoids and biological agents are adopted according to clinical conditions for moderate and severe cases. Budesonide is recommended for mild Crohn’s disease (CD), while potent agents such as biological drugs are preferred for early intervention in moderate and severe CD (92, 93).With respect to the neurological dysfunction in ASD, current established clinical treatments are primarily based on behavioral rehabilitation training, combined with language and social communication training and sensory integration training, which can improve core behavioral symptoms of ASD (94, 95). However, specific etiology-targeted therapies remain largely at the stage of animal experimentation or preclinical inference. For example, animal studies have suggested that microglial modulators such as minocycline may be used to suppress central neuroinflammation in animal models (96). Additional experimental or preclinical approaches will be discussed in the following treatment section.

### 3.2 Treatment of IBD-ASD comorbidity from the perspective of gut microbiota

As discussed above, both the separate onset of IBD and ASD and their comorbidity are associated with gut microbiota dysbiosis. Therefore, the therapeutic focus of this review is to address the treatment of IBD and ASD from the perspective of gut microbiota.

#### 3.2.1 Probiotics and fecal microbiota transplantation

Alterations in gut microbiota composition and decreased abundance of beneficial bacteria are major causes of gut microbiota dysbiosis. Targeting intestinal microorganisms is a crucial therapeutic strategy for patients with IBD, ASD, and those suffering from both comorbid diseases (96–98). Based on published animal experiments and limited preliminary human studies, the anti-inflammatory and immunomodulatory effects of probiotics have been investigated for years. Supplementing tryptophan-metabolizing specific probiotics such as certain strains of Bifidobacterium and Lactobacillus can restore healthy intestinal microbial metabolism, correct the metabolic flux of tryptophan, reduce the accumulation of harmful metabolites, and facilitate the production of beneficial metabolites simultaneously (99). Furthermore, animal studies have found that the commensal gut bacterium Faecalibacterium can regulate indole-AhR signaling, modulate immune homeostasis, and effectively improve autism-related behavioral abnormalities in mice (73).

In recent years, numerous studies have focused on genetically engineered beneficial bacteria that overexpress catalase and superoxide dismutase to construct recombinant strains for the treatment of intestinal inflammation (100, 101). Other research has demonstrated that coating the surface of Escherichia coli Nissle 1917 with nanostructures and sodium alginate layer by layer can enhance the resistance of modified probiotics to oxidative and inflammatory stress (102).

Other studies have revealed that programmable probiotics encapsulated in double-layer microcapsules are capable of remodeling tryptophan metabolism. They can elevate the production of microbial metabolites IAA and IPA, improve intestinal permeability, alleviate inflammation, and inhibit pathogenic strains such as Morganella-Shigella, thereby restoring gut microbiota homeostasis. This approach can effectively prevent inflammatory bowel disease in mice. This approach is cost-effective and holds promise for further clinical translation (103).

Fecal microbiota transplantation (FMT) may have potential therapeutic value for patients with IBD, ASD, and those with comorbid conditions (104–107). A systematic review summarizing relevant RCTs suggested that FMT may improve clinical and endoscopic remission in active ulcerative colitis, but found insufficient evidence for its use in maintenance of remission in UC, or in induction or maintenance of remission in CD. The review concluded that FMT remains an exploratory therapy for IBD and cannot yet be recommended as a routine clinical treatment (105). To date, no clinical studies have specifically evaluated the efficacy of FMT in patients with IBD-ASD comorbidity. However, case reports have shown that FMT can improve gastrointestinal and behavioral symptoms in ASD patients with comorbid gastrointestinal dysfunction (106). It should be noted that ASD patients with gastrointestinal dysfunction are not equivalent to those with IBD-ASD comorbidity, and isolated case reports do not warrant immediate clinical application of FMT. Nevertheless, the observed therapeutic effects of FMT in IBD patients alone, as well as in ASD patients with gastrointestinal symptoms, suggest that FMT may have potential therapeutic value for the comorbid population. In addition to alleviating ASD symptoms, animal studies have demonstrated that prenatal intervention with Lactobacillus can prevent the onset of ASD-related symptoms (107).

The aforementioned therapeutic effects of probiotics and fecal microbiota transplantation are mostly derived from animal experiments or limited preliminary human studies. Large-scale cohort studies and clinical trials are still lacking to validate their clinical applicability. Future clinical research is needed to further define the value of these intervention strategies in patients with comorbid conditions.

#### 3.2.2 Intestinal barrier repair

As discussed above, patients with IBD-ASD comorbidity are likely to have intestinal barrier damage. Currently, clinically available options include glutamine (108), zinc preparations (109) and intestinal mucosal protectants can be applied to repair injured intestinal epithelium (110) and ameliorate intestinal leakage, and can also help modulate immune dysregulation (111). These interventions can block the entry of abnormal tryptophan metabolites and pro-inflammatory factors into the bloodstream, thereby preventing central nervous system damage mediated by the gut-brain axis, and also alleviate the adverse retrograde regulation of intestinal lesions induced by central inflammation.

Currently, clinical approaches to intestinal barrier repair rely primarily on suppressing inflammation or directly enhancing the intestinal mucosal barrier. However, long-term immunosuppressive therapy carries risks of infections and malignancies. Therefore, therapeutic strategies that achieve mucosal healing without immunosuppression—such as targeted tissue regeneration—have attracted increasing attention in recent years. For example, growth factors have been used to treat short bowel syndrome, but their application in inflammatory bowel disease (IBD) has not yet been established. Organoid culture and stem cell-based approaches, which generate *de novo* intestinal tissue, hold promise as novel strategies for restoring intestinal barrier function in patients with IBD (112). However, these therapeutic approaches have not yet been established as routine treatment options for IBD-ASD comorbidity.

#### 3.2.3 Dietary and nutritional interventions

Gut microbiota dysbiosis is an important factor for IBD-ASD comorbidity. Some views suggest that improving gut microbiota dysbiosis through dietary and nutritional interventions may relieve clinical symptoms and slow down disease progression (113) Comorbid patients may adopt prebiotic-supplemented diets. A small-sample preliminary clinical trial suggested combined application of probiotics and moderate-carbohydrate diets can effectively alleviate ASD symptoms, accompanied by corresponding alterations in intestinal microbiome and metabolome profiles (114).

Recent research evidence indicates that nutritional supplements can serve as adjuvant therapies for patients with ASD and those complicated with gastrointestinal disorders, including vitamins, minerals, polyunsaturated fatty acids and antioxidants (115, 116). Increasing intake of anti-inflammatory diets while avoiding pro-inflammatory diets high in sugar and fat can reduce the abnormal regulation of tryptophan-metabolizing enzymes induced by intestinal inflammation (117). In addition, supplementation with coenzymes required for tryptophan metabolism -such as vitamin B6 and vitamin B12 -can lower serum 5-HT levels and relieve abnormal intestinal peristalsis and neurotransmitter deficiency (115, 118).

Although these studies did not specifically target patients with ASD or ASD with comorbid IBD, the therapeutic rationale is based on the notion that nutritional interventions can modulate tryptophan metabolism. Therefore, in light of our previous mechanistic inferences regarding IBD-ASD comorbidity, we suggest that nutritional interventions may also play a role in the treatment of patients with this comorbid condition.

### 3.3 Regulation of synthesis of key enzymes in tryptophan metabolic pathways

Within the mechanistic framework we have proposed for IBD-ASD comorbidity, we consider that tryptophan metabolic disturbances represent an important metabolic process that cannot be overlooked. Key enzymes involved in the kynurenine pathway and 5-HT pathway occupy vital positions in the comorbid pathogenesis, and modulating their activity may have therapeutic potential for improving tryptophan metabolic disturbances. As the core enzyme mediating tryptophan shunting toward the kynurenine pathway, indoleamine 2,3-dioxygenase (IDO) is abnormally activated in both intestinal inflammation of IBD and neuroinflammation of ASD. Current basic studies have verified that specific IDO inhibitors such as 1-methyltryptophan can reduce the production of neurotoxic metabolites including kynurenine and quinolinic acid (119). However, because this therapeutic approach has not yet been validated by large-scale animal model experiments or clinical pilot studies, it still has considerable limitations. With further experimental and theoretical refinement, IDO-specific inhibitors may in the future be applicable to patients with IBD-ASD comorbidity, so as to ameliorate systemic inflammation and alleviate the social and stereotyped symptoms of ASD. Although the mechanistic hypothesis inferred above from basic and clinical observational studies suggests that kynurenine metabolism may play an important role in the pathogenesis of ASD, a recent review that included randomized, placebo-controlled trials (RCTs) evaluating the efficacy of various immunomodulatory and anti-inflammatory drugs for ASD concluded that current RCT evidence does not support the routine use of anti-inflammatory or immunomodulatory agents for the treatment of core ASD symptoms. The review also recommended that future studies should prioritize the selection of patient subgroups with inflammatory phenotypes in trial design to enable further evaluation (120).

Collectively, key human, *in vitro* and animal studies describing the tryptophang‑metabolism-mediated crosstalk between IBD and ASD are summarized in **Table 2**.

**Table 2:** Summary of main human, *in vitro* and animal studies investigating the association between IBD‑ASD comorbidity and tryptophan metabolism.

Research topic category	Study type	Study model and Subjects	Direction of metabolite alteration	Major findings	References
Epidemiological association between IBD and ASD	human observational studies	Multiple nationwide cohorts, meta-analysis of observational studies	Not detected	Positive association between IBD and ASD; higher IBD prevalence in ASD patients; childhood-onset IBD elevates risk of ASD-related neuropsychiatric phenotypes	(3–6), (17)
Peripheral Trp-Kyn pathway alteration in IBD	human observational studies	IBD patients, non-IBD control subjects	Trp ↓;KYN ↑	Altered circulating Trp and abnormal KYN metabolism in IBD patients; intestinal inflammation-induced IDO overexpression depletes Trp	(23), (62)
Peripheral Trp-Kyn pathway alteration in ASD	human observational studies	ASD patients, neurotypical controls	Trp −; KYN ↑; KYNA ↓; QA ↑	Aberrant KYN pathway metabolism in autistic individuals; decreased serum KYNA in children with ASD	(13), (65), (69), (75)
5-HT metabolic abnormality in IBD	human observational studies	IBD patients and healthy controls	5-HT ↑	Changed 5-HT signaling components in IBD patients and their healthy first-degree relatives	(64)
5-HT metabolic abnormality in ASD	human observational studies	ASD patients, control population	5-HT ↑	Hyperserotonemia in autistic patients; peripheral 5-HT fails to cross the blood‑brain barrier	(66), (77, 78)
Gut microbiota regulating Trp-serotonin metabolism *in vitro*/animal studies	*in vitro*/animal studies	Enterochromaffin-like cells, intestinal epithelial cell lines, bacterial monoculture	Trp metabolites indoles variable; 5-HT ↑	Certain gut microbiota directly generate Trp catabolites; some strains produce indoles	(33), (40), (44), (50)
Gut-microbiota-mediated Trp metabolic disturbance	animal studies	Multiple mouse models (colitis model, ASD-like mouse model, germ-free mouse)	Trp ↓; Indole metabolites ↓; 5-HT ↑	Gut dysbiosis disrupts Trp metabolites; microbiota transfer rescues ASD-like behaviors	(81, 82) (98)
AHR-IDO signaling linking gut inflammation and neuro-behavior	animal studies/*in vitro* studies	CD4+ T-cell, astrocyte, colitis mice, neurodevelopmental disorder mouse	KYN ↑; Microbial indoles ↓	Trp‑derived metabolites maintain intestinal immune homeostasis and modulate central neuroinflammation via the AhR‑IDO axis; maternal gut dysbiosis remodels fetal neurodevelopment	(15), (48), (51, 52)
Probiotics/microbiota-targeted intervention	animal studies	DSS-induced colitis mice, BTBR ASD-like mice, 16p11.2 microdeletion mouse	IPA ↑; KYNA ↑	Specific probiotic strains and Faecalibacterium hominis reshape gut microbiota, restore Trp metabolite balance, and ameliorate ASD‑like behaviors and colonic injury in animal models	(73, 74), (104), (107)
Microbiota-transfer/dietary intervention	human observational studies/clinical trials/case reports	ASD children, IBD patients, open-label microbiota transfer cohort	Part of indole-derived metabolites ↑	Open‑label microbiota transfer relieves gastrointestinal and partial autistic symptoms; nutritional/probiotic interventions yield heterogeneous effects, lacking solid clinical evidence for IBD‑ASD comorbidity	(106), (114, (115), (117)

↑, increased level; ↓, decreased level; −, no significant change; Not detected, no statistically significant alteration found; Variable, inconsistent metabolite changes across different studies. IBD, inflammatory bowel disease; ASD, autism spectrum disorder; Trp, tryptophan; Kyn, kynurenine; KYNA, kynurenic acid; QA, quinolinic acid; 5-HT, 5-hydroxytryptamine; IPA, indole‑3‑propionic acid; AhR, aryl hydrocarbon receptor; IDO, indoleamine 2,3‑dioxygenase.It should be noted that current evidence specifically from confirmed IBD-ASD comorbidity cohorts is largely limited to epidemiological associations. Direct human evidence supporting the mechanistic framework proposed in this review remains scarce; most of the mechanistic inferences are derived from animal models, in silico analyses, or studies conducted in separate IBD or ASD populations. Accordingly, the human observational data summarized in this table are primarily derived from epidemiological studies of IBD-ASD comorbidity and from separate mechanistic studies in IBD or ASD cohorts. The limitations of extrapolating these findings to the comorbid context are further discussed in the Limitations section of the main text.

## 4 Conclusions and future perspectives

By analyzing and summarizing the pivotal roles of tryptophan metabolism, the gut-brain axis, and gut microbiota in the pathogenesis of IBD and ASD, we infer that microbiota dysbiosis may act as a permissive factor that disrupts the balance of intestinal tryptophan metabolism, thereby leading to intestinal barrier injury and intestinal inflammation in patients. Under the bidirectional regulation of the gut-brain axis, intestinal barrier damage and intestinal inflammation are progressively aggravated, while disordered tryptophan metabolism breaks intestinal immune homeostasis and further amplifies intestinal inflammatory responses, and may also contribute to worsening neuroinflammation and central nervous system damage in ASD patients. while The bidirectional crosstalk between the intestine and central nervous system mediated by the gut-brain axis forms a vicious cycle involving intestinal barrier dysfunction and immune imbalance, which may ultimately contribute to the pathogenesis of comorbid IBD in patients with ASD.

This review has several limitations. First, it focuses on the single pathway of tryptophan metabolism and does not address the synergistic effects of other abnormalities such as amino acid metabolic disturbances or epigenetic modifications in the comorbidity of IBD and ASD. Second, the evidence supporting the mechanistic hypothesis proposed in this review is derived predominantly from animal models or *in vitro* experiments, with limited direct clinical evidence from patients with IBD-ASD comorbidity; moreover, animal models cannot fully replicate the complex pathological processes of human comorbidity. Third, considerable heterogeneity exists across studies regarding age, sex, dietary habits, medication use, and antibiotic exposure, and these factors have often not been analyzed as major stratification variables, which may introduce bias. Fourth, definitions and assessment criteria for IBD disease activity and ASD phenotypes vary across studies, complicating cross-study comparisons. Fifth, differences in sample types, metabolomic platforms, and microbiome sequencing methods across studies may reduce the comparability of metabolite concentration and microbiota composition data. Sixth, there is currently no unified standardized definition of IBD-ASD comorbidity; inconsistent diagnostic and inclusion criteria for comorbid patients across studies, as well as a lack of standardized criteria for the temporal sequence of comorbidity, limit direct comparisons and the generalizability of conclusions.

Future validation of the mechanistic hypothesis proposed in this review will require further studies and the establishment of unified quantitative standards. At the mechanistic level, future research should move beyond a single metabolic pathway and integrate multi-omics approaches to systematically assess the synergistic pathogenic roles of tryptophan metabolism with other amino acid metabolic abnormalities and epigenetic modifications. At the evidence level, large-scale, multicenter prospective cohort studies are needed, with unified inclusion criteria for comorbid patients—including IBD disease stage and ASD severity grade—and stratified validation across different comorbid subtypes to enhance the generalizability of the hypothesis. At the methodological level, standardized protocols for tryptophan metabolite detection and sample types should be established, and evaluation indices for gut-brain axis function—such as intestinal barrier permeability and neuroinflammatory markers—should be standardized to precisely quantify the impact of tryptophan metabolic disturbances on the gut-brain axis. Ultimately, future research should develop precision-targeted therapeutic strategies based on the individualized metabolic profiles and microbial characteristics of comorbid patients, with the goal of simultaneously correcting metabolic disturbances and alleviating symptoms of both diseases.
